# Factors associated with intention to smoke cigarettes among never smoker school going adolescents in Zambia

**DOI:** 10.4314/ahs.v23i1.63

**Published:** 2023-03

**Authors:** Paul Syapiila, David Mulenga, Mazyanga Mazaba, Erick Njunju, Cosmas Zyambo, Gershom Chongwe, Seter Siziya

**Affiliations:** 1 The Copperbelt University School of Medicine, Public Health; 2 The Copperbelt University School of Medicine, Clinical Sciences; 3 Zambia Ministry of Health, The Health Press, Zambia National Public Health Institute; 4 The Copperbelt University School of Medicine, Basic Sciences; 5 University of Zambia School of Public Health, Community and family health; 6 Tropical Diseases Research Centre

**Keywords:** Intention, smoke, cigarettes

## Abstract

**Background:**

Cigarette smoking intention is a strong predictor of cigarette smoking initiation. There is limited data on predictors of cigarette smoking intentions among adolescents in developing countries.

**Objective:**

To determine factors associated with cigarettes smoking intentions among never-smoked adolescents.

**Methods:**

The study utilized the Zambia 2011 Global Youth Tobacco Survey dataset on adolescents.

**Results:**

Being in grade nine compared to grade seven (AOR 0.43, 95%CI 0.23-0.82). Having a smoking father (AOR 2.38, 95%CI 1.25-453) mother (AOR 11.77, 95%CI 4.16-33.33), or both parents (AOR 7.05, 95%CI 2.91-17.10) showed significantly higher chance of having smoking intentions than having non-smoker parents. Also, having some (AOR 1.97, 95%CI 1.12-3.47), most (AOR 5.37, 95%CI 2.82-10.25), or all (AOR 3.75, 95%CI 1.64-8.56) smoker close friend was significantly associated with smoking intention compared to having none-smoker friends. Being around others who smoked in out-door places 1-2 days (AOR 2.16, 95%CI 1.19-3.93), 5-6 days (AOR 3.21, 95%CI 1.51-6.83) and 7 days/week (AOR 2.73, 95%CI 1.41-5.30) were also associated with one's intention to smoke cigarettes compared to not being around smokers in outdoor public places 7 days/week.

**Conclusion:**

Having smoking parents, smoking friends or around people who smoke in public places were associated with cigarette smoking intentions among adolescents.

## Introduction

Tobacco smoking is one of the major public health challenges and the leading risk factor for non-communicable diseases globally^1^. World Health Organization estimates that tobacco kills more than 8 million people each year, which is about half its users and bout 80% of these users reside in low and middle income countries^2^. Globally, cigarette smoking prevalence among adolescents aged 13 to 15 years is estimated around 6.5% distributed as 8.9% among boys and 3.8% among girls. America and Europe have the highest smoking prevalence among adolescents at 8.7% with Eastern Mediterranean region lowest at 4.7%^3^.

African region is at its early stages of tobacco pandemic with no robust interventions informed by evidence. Recent studies in Africa shows that tobacco use prevalence ranges between 4.6% to 36.6% among girls and 7.8 to 36.5% among boys^2^. World Health Organization estimates cigarettes smoking prevalence in Africa at 6.2% (8.5 among boys and 3.7 among girls) among adolescents^3^. Jallow et al also reported 4.5% and 16.7% of adolescents in Gambia as current smoker and ever smoker's respectively^4^. With the rapid population growth in Africa, an increase in consumer purchasing power and increased efforts by the tobacco industry to enlarge their market in the region, Africa is experiencing an increase in tobacco use. If appropriate interventions are not put in place, WHO estimates deaths due to tobacco use in Africa to double by the year 2030^2^.

Cigarette smoking trends in Zambia increases with the increase in age from 2.9% for example in age group 15-19 years to 31.9% in the age group 50-59 years among males^5^. The Zambia Demographic and Health surveys (2001-2002, 2007, 2013-14 and 2018 have also demonstrated that people in rural parts of the country and the poor dispassionately have higher prevalence of cigarette smoking than their counterparts in urban areas^5^–^8^. Although the prevalence of tobacco smoking increases with age, it has been noted that most people start smoking while still young^2^. Muula & Siziya reported 40.1% as ever smokers while Zyambo et al reported 7.8% among boys and 5.8% among girls as current smokers among adolescents in school^9^,^10^. The figures may be on increase as the tobacco industry increase its campaign strategies towards adolescents in the middle and low income countries^11^.

Determinants of intention to perform a behavior are very important in predicting future behavior thereby helping in coming up with evidence-based interventions. A number of risk factors for the intention to smoke tobacco cigarettes have been reported. These include poor knowledge of its harmful effects, having a close friend or family members who smoke, exposure to people who smoke in public places, stress, positive attitude towards smokers and lack of family smoking restrictions. Mohammadproorals et al for example reported general risk-taking behavior, having a positive attitude toward cigarettes smoking and having a family member who smoked as risk factors for intention to smoke tobacco cigarettes. They however did not find gender or age to be associated with smoking intentions^12^. To the contrary, Wu et al found being male, together with having a smoking mother and education level of mother as predictors of intention to smoke^13^. Hock et al also did not find gender or having a family member who smoked to be associated with smoking intention^14^. However, in line with the findings of Mohammadproorals et al and Wu et al findings, Patino-meso et al^15^ reported the smoking status of a family member as a predisposing factor for smoking intentions. Hock et al however found smoking friend, social norm and poor knowledge of the ill health effects of smoking to be predictors of smoking intentions.

The importance of knowing factors associated with intention to smoke cigarettes cannot be over emphasized as intention is a strong predictor of a future behavior as has been shown by behavioral theories like the theory of reasoned action^16^ and the theory of planned behavior^17^. The theory of reasoned action for example assumes, that the greater the intention to perform a behavior, the greater likelihood of actually performing that behavior. Behavior intention is the most proxy predictor of behavior, which is influenced by the adolescent's attitude towards behavior, and the subjective norms. The relationship between intention to smoke and behavior action of smoking is not perfect but intention is significantly a predictor of adolescents smoking behavior^18^

There is scarcity of information on predictors of intention to smoke cigarettes among the school going adolescents in Zambia. This study therefore aimed determining risk factors associated with the intention to cigarette smoking among Zambian adolescents. The findings from this study have provided insight into the formulation of locally tailored policies toward prevention and control of cigarette smoking among adolescents in Zambia.

## Methodology

### Data source sample size and sampling

This study utilized the Zambia 2011 Global Youth Tobacco Survey (GYTS) data set. This was a school-based survey which collected data on the prevalence of tobacco use, intention to use as well as a number of possible associated factors among grades 7, 8 and 9 in Zambia. GYTS uses a global standard methodology that utilizes a two-stage cluster sampling design in the selection of a nationally representative sample. At the first stage, schools were selected proportional to enrolment size from the list of both private and public schools in the country. Classes at selected schools were then randomly selected and all pupils in the selected classes were eligible to participate in this study.

Global Youth Tobacco Surveys uses the sample size of at least 1,500 student responses and a minimum of 20 schools for each domain^19^. Therefore, with an estimated 80% response rate for schools and as well as students, at least 25 schools and 1750 students are selected for participation in the study for each domain. Depending on the number of required domains, the total required responses therefore range from 1,500 to 10,000 students. The required sample size is based on following indicators of statistical quality that have been established by GYTS findings. Data is then collected through the use of the standard core questionnaire which has a set of optional questions that allows adaptation by individual countries to meet their need.

A total sample size of about 6,063 was required for this survey. PC sample, a sampling software developed for CDC youth surveys was used for both school and class sampling. An overall response rate of 55.7% was achieved during the survey giving a total of 3,377 student responses^31^. IBM SPSS statistics version 21 software package was then used to describe and analyse the data for this study.

### Measures

**Primary outcome:** The primary outcome was intention to smoke cigarettes among those who never smoked cigarettes as defined by variable CR15 (Q19) “At any time during the next 12 months, do you think you will smoke a cigarette?” and if they had never smoked cigarettes as defined by variable CR35 (Q40) “How long ago did you stop smoking?” Those who answered definitely not or probably not were considered to have no intention to smoke while those who answered definitely yes and probably yes were considered to have intentions to smoke cigarettes during the next 12 months.

**Independent variables:** A total of three demographic variables and six risk factors were included for analysis in this study. These were based on the following questions of the Zambia 2011 Global Youth Tobacco Survey data set: How old are you?, What is your sex?, In what grade/form are you?, Do your parents smoke?, Do any of your closest friends smoke cigarettes?, Do you think the smoke from other people's cigarettes is harmful to you?, During the past 7 days, on how many days have people smoked in your home or in your presence?, During the past 7 days, on how many days have people smoked in your presence, in enclosed public places?, and During the past 7 days, on how many days have people smoked in your presence, in outdoor public places?.

**Statistical analysis:** All the independent variables were then run against the primary outcome in bivariate logistic regression. Independent variables which were significantly associated with the primary outcome in bivariate logistic regression analysis (p-value ≤ 0.05) were then included in the stepwise forward logistic regression analysis to determine their non-confounded association with intention to smoke cigarettes among those who had never smoked. Hosmer-Lemeshow test was used to test the best ‘Fit’ model. Crude and adjusted odds ratios with their 95% confidence intervals and p-values have been reported for the final model. Possible interactions between the two most significant predictor variables were also analysed.

## Results

### Descriptive statistics

Of the 3,377 pupils in grades 7, 8 and 9 who took part in the survey, 3,296 (97.6%) had responses for both question 40 (CR35) and question 19 (CR15) while 81 (2.4%) had missing responses to either question. And out of the 3296 with responses to both questions, only 2,570 (78.0%) had never smoked cigarettes and were thus considered in this study. Of those who had never smoked cigarettes 4.1% (106) had intention to smoke in the next 12 months.

Table 1 shows that most of the participants in this study were aged between 13 years and 16 years with males tending to be slightly older than females. The distribution of all demographic variable was similar between male and female participants.

**Table 1:** Social demographic characteristics of study participants by gender

Social demographic factors	Males (%)	Females (%)	Total (%*)
**Age of pupils (years)**			
< 12	28 (1.7)	59 (3.5)	87 (2.6)
12	96 (6.0)	134 (8.0)	230 (7.0)
13	248 (15.4)	274 (16.4)	522 (15.9)
14	320 (19.8)	401 (23.9)	721 (21.9)
15	325 (20.1)	367 (21.9)	692 (21.0)
16	326 (20.2)	276 (16.5)	602 (18.3)
17+	270 (16.7)	164 (9.8)	434 (13.2)
**Total**	**1613(49.1)**	**1675 (50.9)**	**3288 (100)**
**Pupil's grade**			
7	601 (37.3)	632 (38.1)	1213 (37.7)
8	521 (32.4)	556 (33.6)	1077 (33.0)
9	488 (30.3)	469 (28.3)	957 (29.3)
**Total**	**1610 (49.3)**	**1657 (50.7)**	**3267 (100)**
**Intention to smoke**			
Yes	46 (3.7)	54 (4.1)	100 (3.9)
No	1184 (96.3)	1251 (95.9)	2435 (96.1)
**Total**	**1230 (48.5)**	**1305 (51.5)**	**2535 (100)**

* Indicate column percentage.

### Association of study variables with intention to smoke cigarettes

In bivariate analyses of demographic variables (table 2): Gender and Age were both not significantly associated to intention to smoke. They were, therefore, not considered in the multivariate analysis. Participant's grade was however significantly associated with intention to smoke cigarettes and was included in multivariate analysis.

**Table 2:** Bivariate associations of social demographic and other risk factors and intention to smoke

Factors	Intention to smoke		Crude OR (95% CI)	P-value
	Yes (%)	No (%)		
**Age of pupils (years)**				
< 12	3 (5.1)	56 (94.9)	1	
12	6 (3.1)	187 (96.9)	0.60 (0.15–2.47)	0.479
13	10 (2.4)	412 (97.6)	0.45 (0.12–1.70)	0.240
14	19 (3.4)	544 (96.6)	0.65 (0.19–2.27)	0.502
15	18 (3.4)	517 (96.6)	0.65 (0.19–2.28)	0.500
16	31 (6.8)	423 (93.2)	1.37 (0.41–4.62)	0.614
17+	18 (5.5)	307 (94.5)	1.09 (0.31–3.84)	0.888
**Sex of pupil**				
Male	46 (3.7)	1184 (96.3)	1	
Female	54 (4.1)	1251 (95.9)	1.11 (0.74–1.66)	0.607
**Pupil's grade**				
7	55 (5.9)	879 (94.1)	1	
8	32 (3.8)	800 (96.2)	0.64 (0.41–1.00)	0.049
9	16 (2.1)	748 (97.9)	0.34 (0.19–0.60)	< 0.001
**Smoking parent**				
None	34 (2.0)	1701 (98.0)	1	
Father only	21 (6.4)	305 (93.6)	3.45 (1.97–6.02)	<0.001
Mother only	7 (22.6)	24 (77.4)	14.59 (5.89–36.17)	<0.001
Both	9 (16.4)	46 (83.6)	9.79 (4.44–21.59)	<0.001
**Smoking closest friend**				
None	38 (2.1)	1787 (97.9)	1	
Some of them	30 (6.2)	455 (93.8)	3.10 (1.90–5.06)	<0.001
Most of them	22 (16.4)	112 (83.6)	9.24 (5.28–16.15)	<0.001
All of them	12 (13.2)	79 (86.8)	7.14 (3.59–14.20)	<0.001
**Think smoke from others is** **harmful to them**				
Definitely not	31 (4.2)	702 (95.8)	1	
Probably not	22 (7.9)	255 (92.1)	1.95 (1.11–3.44)	0.020
Probably yes	20 (6.1)	309 (93.9)	1.47 (0.82–2.61)	0.195
Definitely yes	28 (2.4)	1157 (97.6)	0.55 (0.32–0.92)	0.023
**Around others who smoke at** **home**				
0 days/ week	51 (2.7)	1839 (97.3)	1	
1 to 2 days/ week	17 (6.4)	249 (93.6)	2.46 (1.40–4.33)	0.002
3 to 4 days/ week	11 (9.2)	109 (90.8)	3.64 (1.84–7.18)	<0.001
5 to 6 days/ week	10 (10.4)	86 (89.6)	4.19 (2.06–8.54)	<0.001
7 days/ week	15 (8.2)	167 (91.8)	3.24 (1.78–5.88)	<0.001
**Around others who smoke in** **enclosed public places**				
0 days/ week	49 (3.0)	1582 (97.0)	1	
1 to 2 days/ week	18 (4.3)	399 (95.7)	1.46 (0.84–2.53)	0.181
3 to 4 days/ week	16 (10.3)	139 (89.7)	3.72 (2.06–6.71)	0.001
5 to 6 days/ week	4 (3.4)	114 (96.6)	1.13 (0.40–3.20)	0.814
7 days/ week	17 (7.6)	208 (92.4)	2.64 (1.49–4.67)	0.001
**Around others who smoke in** **outdoor public places**				
0 days/ week	38 (2.5)	1481 (97.5)	1	
1 to 2 days/ week	22 (5.1)	410 (94.9)	2.09 (1.22–3.58)	0.007
3 to 4 days/ week	10 (5.7)	165 (94.3)	2.36 (1.16–4.83)	0.018
5 to 6 days/ week	12 (8.5)	130(91.5)	3.60 (1.84–7.05)	<0.001
7 days/ week	17 (6.8)	232(93.2)	2.86 (1.59–5.14)	<0.001

Having no parent or friend who smoke showed significantly lower chances of having smoking intentions compared to having a father, mother and both parents or some, most and all friends who are smokers respectively. Those who thought smoke from others was probably not harmful to them had significantly higher chance of having intention to smoke while those who thought it was definitely harmful had lower chances compared to those who thought it definitely had no harmful effects. Being around others who smoked at home, in enclosed public places and in outdoor public places were also seen to be associated with intention to smoke in bivariate analysis (p-value <0.05). All these variables were thus included in multivariate analysis to determine predictors of intention to smoke.

From Table 3, grade nines (AOR 0.43, 95% CI 0.23-0.82) were seen to be less likely to have smoking intentions compared to grade sevens and this was statistically significant. Having a father (AOR 2.38, 95%, CI 1.25-4.53), a mother (AOR 11.77, 95% CI 4.16-33.33) or both parents AOR 7.05, 95% CI 2.91-17.10) who smoked were shown to significantly have higher chance of having intention to smoke cigarettes compared to participants with no parent who smoked. Also, having some of close friends (AOR 1.97, 95% CI 1.12-3.47), most of the close friends (AOR 5.37, 95% CI 2.82-10.25), or all close friends (AOR 3.75, 95% CI 1.64- 8.56) who smoked, were also seen to be significantly associated with cigarettes smoking intention among the adolescents. Although being around others who smoke in outdoor public places for 3 to 4 days in a week did not show increased intention to smoke cigarettes that was statistically significant compared to those with 0 days/ week AOR=1.87, 95%CI=0.79-4.44), being around others who smoke in outdoor public places for 1-2days/ week (AOR 2.16, 95%CI 1.19-3.93), 5-6 days/week (AOR 3.21, 95%CI 1.51-6.83) and 7 day/ week (AOR 2.73, 95%CI 1.41-5.30) had significantly higher levels of intention to smoke than those with 0 days/ week.

**Table 3:** Factors independently associated with intention to smoke among Zambian adolescents

Independent factors	Adjusted Odds Ratios	95% CI		P-value
		Lower limit	Upper limit	
**Pupil's grade**				
7	1			
8	0.75	0.45	1.24	0.261
9	0.43	0.23	0.82	0.010
**Smoking parents**				
None	1			
Father only	2.38	1.25	4.53	0.008
Mother only	11.77	4.16	33.33	<0.001
Both	7.05	2.91	17.10	<0.001
**Smoking closest friend**				
None	1			
Some of them	1.97	1.12	3.47	0.018
Most of them	5.37	2.82	10.25	<0.001
All of them	3.75	1.64	8.56	0.002
**Around others who smoke in** **outdoor public places**				
0 days/ week	1			
1 to 2 days/ week	2.16	1.19	3.93	0.011
3 to 4 days/ week	1.87	0.79	4.44	0.158
5 to 6 days/ week	3.21	1.51	6.83	0.002
7 days/ week	2.73	1.41	5.30	0.003

However, thinking smoke from others was harmful to oneself, being around others who smoke at home, and being around others who smoke in enclosed public places were all not independently associated with the intentions to smoke cigarettes in this study. The Hosmer-Lemeshow X2 for the model was at 11.439, p-value 0.178, indicating no evidence of poor fit for the model.

All possible two-way interactions between predictor variables in the final model were examined and none of the interactions were significant. They were thus none reported in this article

## Discussion

Smoking intention precedes smoking experimentation and thus initiation. It is therefore a strong predictor of tobacco smoking. Interventions directed at this stage are very beneficial at preventing adolescent from initiating the smoking habit. Prevalence of cigarette-smoking intentions among never-smoker adolescents in this study was found to be 4.1%. This is slightly lower than what was reported in Nigeria (6.3%) and China (6.9)^20^,^21^. Mohammadpoorasl et al however reported similar prevalence of 5.0%^22^. These differences could be due the difference in the prevalence of influencing factors like smoking friends or relatives and social norms. It could also be due to differences the classification of those with smoking intention or the population under consideration like never smokers vs. current non-smokers.

A number of studies have been done to determine factors associated with intention to smoke cigarettes^19^–^26^. In this study, having a parent or a close friend who smokes was significantly associated with the intention to smoke cigarettes. A number of similar studies have also found that those with close relatives or friends who smoked were more likely to have smoking intentions compared to those who did not have any^12^,^14^,^15^,^21^,^22^. This is in line with Bandura's observation that people generally learn from those close to them through observation and imitation^23^. This is also supported by the theory of reasoned action which states that normative belief determines intention16. Children whose close peers, relatives or other role models smoke are more likely to develop a positive perception and have intentions to smoke cigarettes. This is because the expectation of important persons around an individual determines what is perceived to be normal and thus intention.

This study also revealed that having a mother who smoke had higher odds of having intention to smoke than having a father who smoke although both were significant predictors of intention to smoke. Wu et al however did not find smoking habits of fathers to have a significant effect on the smoking intentions of their children in a study among Chinese college students although that of their mothers did^13^. This was attributed to the fact that mothers traditionally spend more time with children in Chinese societies than fathers. Mothers therefore tended to have a stronger influence in the development, attitude and behavior of their children than fathers. This could therefore explain why adolescents with mothers who smoked where more likely to have smoking intentions than those with fathers who smoked in this study since even in the Zambian culture, mothers tend to spend more time with their children than fathers.

In a study in Madagascar by Veeranki et al among school going adolescents, exposure to second-hand smoking in outdoor public places was found to be significantly associated with the intention to smoke^24^. Exposure to second-hand smoking may not only develop a positive perception and attitude towards smoking among adolescents as was reported by Cremers et al 25, but could also make one get used to the smoke thereby developing smoking intentions among never-smokers. It is also a risk factor for ill health and death^2^. Being with smokers in enclosed public places and at home were not found to be independently associated with intention to smoke in this study. This could be due to the possibility that exposure to smoke at home is mostly from parents while exposure to smoke in public places was mostly from close friends both of which were independently associated with intention to smoke in the current study. However, being with smokers in outdoor public places was found to be statistically associated with intention to smoke cigarettes among Zambian adolescents similar to the findings of Veeranki et al^24^.

As anti-smoking campaigns get intensified in high income countries, the tobacco industry is also working hard trying to regain their lost income by focusing on middle and low income countries^26^,^27^. Knowing that most decisions to smoke are made before the age of 23 years, they mostly target adolescents in these countries 2, 28, It is therefore important that evidence-based interventions are put in place to protect these adolescents in low and lower middle-income countries. This study also showed that intention to smoke cigarettes among young adolescents develop early in Zambia. Results showed that smoking intentions reduced from grade seven to grade nine with grade nines showing significantly lower odds of intention to smoke than grade sevens. This is contrary to the findings of Wu et al and Mohammadpoorasl et al both of which did not find age to be associated with intention to smoke^12^,^13^. However, Patino-Meso et al reported age-group 10 to 15 to be associated with high smoking onset with the more frequent ages being 12 years at 40% and 13 at 35% corresponding to grades 7 and 8 respectively in a Zambian setup15. This could be explained by the high influence of peer pressure at this age group and also the fact that this is the age group targeted by Tobacco companies. Antismoking interventions could therefore be intensified for grade sevens or lower.

Patino-Meso et al in a study among high school students in Spain reported that perceived low risks of tobacco smoking as one of the factors driving the intention to smoke cigarettes^15^. In our study, we found that those who did not consider second hand smoke as harmful were not at significantly higher likelihood of having intentions to smoke than those who thought it was. This is hard to explain as perceived risk is expected to inform intention. A number of other studies however reported perceived low risk of second-hand cigarette smoke and high risk taking tendencies as risk factors for intention to smoke 12,14,24,29. One way to reduce this low perception of the risks of smoking tobacco could be introducing anti-smoking health lessons early into pupil's school curriculum and intensifying their teaching.

The current study also showed that adolescents who had a smoking mother and most or all friends who smoked had the highest chance of having intentions to smoke cigarettes. This was much higher than the additive effects of the two individual variables. Anti-smoking interventions against cigarette-smoking among adolescents should therefore target both parents and their peers if the fight is to be a success.

## Limitations

The response rate for the initial survey of this study was quite low (55.7%). This could be due a number of reasons like parents not signing the assent forms for their children to participate in the study, high absenteeism resulting in none availability of most selected students during data collection or failure to just answer and return the questionnaire by selected participants. Since we could not establish the reasons for non-response, it remains possible that our results may be biased to the extent that non-response rates differed between those with smoking intention and those without smoking intentions. Therefore, none response bias could not be ruled out. As a result, we could not confidently estimate the prevalence for smoking intentions among the Zambian adolescents in this study. We however, have no reason to believe that non-response rate differed. There was also a limitation in claiming causality between predictor and outcome variables as the study was based on a cross section survey. Also, the findings of the current study may only be true to grades 7, 8 and 9 in Zambia.

## Conclusion

This study has demonstrated that exposure to second-hand cigarette smoke in outdoor public places, exposure through parents and close friends who smoke were associated with the intention to smoke cigarettes among adolescent never-smokers in Zambia. The association was even stronger if the smoking parent was a mother or if all close friends smoked.

## Recommendations

Factors that determine the existence of a given problem may differ from one setting to another. This study has therefore provided us with the exposure to second-hand smoke factors that were associated with the intention to smoke among school going adolescents in the Zambian setting who had never smoked cigarettes. As intention to smoke determines future smoking behaviours, we recommend that lessons on harmful effects of tobacco smoking should be introduced in the school curriculum and their teaching be intensified at primary school level. To yield desired results, interventions should also be directed at family and community levels. They should include registering and enforcing laws that prohibits shops from selling tobacco products to adolescents and smoking in public places, increasing taxes on tobacco products thereby making unaffordable to many, Parents and guardians need to know that their children will learn from what they observe them do through health education programs organized at community level. This cannot be overemphasized considering the potential effects of the organized and experienced tobacco industry tact as they target youths in medium and low-income countries.
